# Selected Metabolites Profiling of *Staphylococcus aureus* Following Exposure to Low Temperature and Elevated Sodium Chloride

**DOI:** 10.3389/fmicb.2020.00834

**Published:** 2020-05-08

**Authors:** Mousa M. Alreshidi

**Affiliations:** Department of Biology, College of Science, University of Ha’il, Hail, Saudi Arabia

**Keywords:** *S. aureus*, metabolic profiling, stress response, purine and pyrimidine bases, food storage

## Abstract

*Staphylococcus aureus* is one of the main foodborne pathogens that can cause food poisoning. Due to this reason, one of the essential aspects of food safety focuses on bacterial adaptation and proliferation under preservative conditions. This study was aimed to determine the metabolic changes that can occur following the exposure of *S. aureus* to either low temperature conditions or elevated concentrations of sodium chloride (NaCl). The results revealed that most of the metabolites measured were reduced in cold-stressed cells, when compared to reference controls. The major reduction was observed in nucleotides and organic acids, whereas mannitol was significantly increased in response to low temperature. However, when *S. aureus* was exposed to elevated NaCl, a significant increase was observed in the metabolite levels, particularly purine and pyrimidine bases along with organic acids. The majority of carbohydrates remained constant in the cells grown under ideal conditions and those exposed to elevated NaCl concentrations. Partial least square discriminate analysis (PLS-DA) of the metabolomic data indicated that both, prolonged cold stress and osmotic stress conditions, generated cells with different metabolic profiles, in comparison to the reference controls. These results provide evidence that, when bacterial cells exposed to low temperatures or high concentrations of NaCl, experience *in situ* homeostatic alterations to adapt to new environmental conditions. These data supported the hypothesis that changes in metabolic homeostasis were critical to the adaptive processes required for survival under alterations in the environmental conditions.

## Introduction

*Staphylococcus aureus* is both a commensal and a notable human pathogen, which causes a wide range of infections in nosocomial and community settings. The ubiquitous nature of this bacterium is partially attributed to its ability to sense changing environmental factors and respond accordingly by adapting to a wide range of environmental alterations (Onyango et al., 2012, 2013; Crompton et al., 2014; Alreshidi et al., 2015, 2016) as well as an impressive ability to resist innate immune defense mechanisms in the host resulting in systemic infections (Haste et al., 2012; Jongerius et al., 2012). However, the mechanisms behind the physiological adaptations of *S. aureus* under a diverse range of hostile conditions are still not clear. It has been hypothesized that changes in the patterns of metabolites and proteins are critical for its adaptation and infection initiation (Alreshidi et al., 2015, 2016). A study has revealed an adaptation mechanism of *S. aureus* under glucose limiting conditions by employed alterations in its metabolites and proteins (Liebeke et al., 2011). It was found that, when *S. aureus* was exposed to even small alterations in environmental conditions, it led to noticeable changes in the amino acid profiles, fatty acids and protein compositions (Crompton et al., 2014; Alreshidi et al., 2016). Therefore, it was concluded that alterations in metabolites and proteins facilitated the survival of *S. aureus* when exposed to confined nutrition in combination with higher concentrations of NaCl. Another study on *S. aureus* demonstrated that on exposure to hydrogen peroxide (H_2_O_2_) combined with various temperatures substantially affected the amino acid metabolites and the consumption of free amino acids in the media in comparison to reference control samples (Murphy et al., 2018). When *S. aureus* was grown at low temperature (4°C), noticeable changes were observed in the structure of cell wall and size of the cells and colonies (Onyango et al., 2012, 2013). Small colony variants (SCVs) are sub-populations of bacterial cells which are characterized by reduced metabolic activity, pigmentation, and virulence factors (Tuchscherr et al., 2010; Proctor et al., 2014). Biofilm is a complex bacterial community embedded within an extracellular matrix adherent to a surface (Arciola et al., 2002; Adnan et al., 2011). SCVs occur during biofilm formation. Enhanced generation of SCVs and biofilm provide bacteria with the advantage of resistance against changes in environmental conditions (Singh et al., 2010; Mirani et al., 2015; Dubois-Brissonnet et al., 2016).

Low temperatures used in refrigeration and high NaCl concentrations are both used as food preserving techniques to prevent bacterial contamination, by making growth conditions sub-optimal, if not inhibitory, for most potential contaminants (Burgess et al., 2016). However, certain bacteria, such as *S. aureus*, have been shown to adapt, survive and even proliferate under these adverse conditions encountered in food production and storage, leading to a restricted shelf life of food products (Carrera et al., 2017). It has also been shown that, a different range of temperatures, combined with various environmental factors also led to altered metabolomic and proteomic responses (Alreshidi et al., 2016). At the bacterial cell level, a temperature decrease reduces the fluidity of cell membranes, which in turn affects active transport and protein secretion (Mykytczuk et al., 2007). Additionally, transcription and translation efficiency is reduced due to stabilization of DNA and RNA secondary structures (Chanda et al., 2010). Protein folding also becomes inefficient and ribosomes need to exhibit adaption for adequate function (Phadtare, 2004). A previous study showed that the exposure of *S. aureus* to low temperature resulted in substantial up-regulation of certain ribosomal proteins, citric acid and a significant reduction in a high number of amino acids (Alreshidi et al., 2015).

*Staphylococcus aureus* is considered to be an osmo-tolerant bacterium (Shebuski et al., 2000; Vilhelmsson and Miller, 2002a, b), however, little is understood about the mechanisms conferring high salt tolerance by the bacteria. Our recent study investigated amino acid metabolites and proteomic responses of *S. aureus* when incubated in a minimal medium supplemented with 5% NaCl. The results indicated significant alterations in amino acid concentrations and protein regulations (Alreshidi et al., 2019). However, to date this experimental design has yet to be adjusted to investigate nucleotides, nucleosides, organic acids and sugar alcohol levels in response to the conditions of low temperature and/or high NaCl. Therefore, this study aims to characterize the metabolic changes (specifically in reference to carbohydrates; pyrimidine and purine bases; nucleosides and nucleotides) that occur as a result of low temperature (4°C) and elevated sodium chloride (5%) conditions. It is hypothesized that the exposure of *S. aureus* to conditions of low temperature and elevated NaCl induce significant alterations in its metabolic profiles.

## Materials and Methods

### *S. aureus* Strain Preservation and Storage

*Staphylococcus aureus* used in the current study was isolated from patients who were suffering from chronic muscle pain (Butt et al., 1998). This bacterium was used in following investigations to study metabolic and proteomic adaptation to alterations in the environmental factors (Onyango et al., 2012, 2013; Alreshidi et al., 2015, 2016). The bacterial strain was grown as culture stock on horse blood agar (HBA) and preserved on sterile glass beads at −80°C with a regular sub-culturing to maintain viability. This bacterium was checked frequently using API^TM^ Staph biochemistry and through the amplification of 16S rRNA gene by polymerase chain reaction (PCR) (Brown et al., 2001).

### Bacterial Growth Conditions at Low Temperature (4°C)

Overnight cultures (50 ml) of *S. aureus* were grown in Tryptic Soy Broth (TSB) at 37°C with constant agitation (120 rpm). Eight flasks containing 95 ml TSB culture media were inoculated with 5 ml of overnight culture in 500 ml conical flasks which were then incubated at 37°C with constant agitation (120 rpm) for 3 h. Four biological replicates were harvested at the mid-exponential phase of growth (3 h) and processed for analyses to represent the control samples. The remaining four cultures were kept at lower temperature of 4°C for 14 days. Cell viability and numbers were tested by the plating method and the identity was confirmed with API. The reference control samples and the cold-treated samples were harvested by centrifugation at 6500 × *g* for 25 min at 4°C. Harvested cells were then washed three times with phosphate buffered saline (PBS) to ensure the elimination of all residual TSB. The washed cells were immediately quenched using liquid nitrogen for lyophilization and subsequent extraction for metabolic analysis.

### Bacterial Growth and Incubation Conditions With Additional of 5% NaCl

Overnight cultures of *S. aureus* were grown in Tryptic Soy Broth (TSB) at 37°C with constant agitation (120 rpm). Eight flasks containing 95 ml TSB culture media were inoculated with 5 ml of overnight culture in 500 ml conical flasks which were incubated at 37°C with constant agitation (120 rpm) for 3 h. Replicate cultures were harvested and washed three times using phosphate buffer saline (PBS) to ensure the elimination of TSB medium. Washed cells were then incubated in a defined minimal medium consisting of PBS with trace as described in Alreshidi et al. (2019). The incubation medium did not allow the cells replication but provided required elements and nutrients bases of energy and nitrogen that help bacterial cells to have active metabolism. The number of cells was assessed by the plating techniques to determine colony forming units (CFU) at initial and final times of incubation for both control and NaCl stressed samples.

### Metabolites Extraction

Washed cells were lysed using snap-frozen and thawing technique 3 times and placed in a dryer machine overnight. Approximately 10–12 mg of lyophilised cells were resuspended with 10 ml of 1:1(v/v) of cold methanol/water stored at −20°C and mixed thoroughly. The methanol/water lyophilised cell slurries were snap frozen in liquid nitrogen and placed in freezer at (−20°C) for 30 min for a process of slow thawing. Metabolites were then separated from the cell debris by centrifugation at 6,500 *× g* for 25 min. The supernatants containing the metabolites were dried using a centrifugal vacuum drier (CentriVap, LABCONCO, VWR) (Alreshidi et al., 2015).

### Metabolites Identification and Quantification

Dried cytoplasmic metabolites including carbohydrates and purine and pyrimidine metabolites were evaluated by forming the methoxy-amine-trimethylsilyl (TMS) derivatives by reacting the dry extracts with Methoxyamine-HCL (MOX) and Bis(trimethylsily) trifluoroacetamide (BSTFA). Lyophilised metabolites were mixed with 50 μl of MOX vortexed very well, and then heated at 60°C for 30 min. Samples were then allowed to cool and subsequently 150 μl of BSTFA was added to each sample, vortexed and heated at 100°C for 60 min. Derivatised samples were analyzed by auto-sampler gas chromatography (Agilent, Hewlett-Packed 5973) coupled with mass spectrometry. The injection volume was 1 μl/sample and flow rate was 0.5 ml/min. Metabolites in the chromatogram were identified on the basis of matching their mass spectra and retention time indices with data in use-generated mass spectral libraries generated from reference standards.

### Metabolic Profile Data Processing and Statistical Analysis

The acquired metabolite data obtained from GC-MS were exported to an Excel^®^ (Microsoft^®^) sheet. The exported data were then imported to STATISTICA (6, StatSoft) (ANOVA) to identify the metabolites that were significantly altered in response to lower temperature and elevated NaCl. Multivariate statistical analysis was then preformed using the partial least squares discriminant analysis (PLS-DA) as implemented in MetaboAnalyst 4.0 on-line package to better identify classification and clustering (www.metaboanalyst.ca) (Xia et al., 2009). The data were preprocessed by the sum normalization, with mean-centered and auto-scaling before creating the model. The model complexity and validity were assessed by cross-validation including the goodness-of-fit parameter (R2) and the goodness-of-predication parameter (Q2).

### Metabolic Pathway and Enrichment Analyses

Metabolic pathway analysis, which combines Metabolite Set Enrichment Analysis (MSEA) and Metabolomic Pathway Analysis (MetPA) was conducted using MetaboAnalyst 4.0 (Xia et al., 2009). GC-MS data were submitted to metaboAnalyst specifically to MetPA and MSEA with annotation based on compound names. Accepted metabolites were manually checked by different databases including KEGG and pubChem databases. The pathway library of *S. aureus* N315 was selected for pathway analysis. Metabolite sets enrichment was preformed according to KEGG database which contains 84 metabolite sets, and at least 2 compounds represent each metabolite sets.

## Results

### Metabolites Analysis at Low Temperature (4°C) for 2 Weeks

**FIGURE 1:**
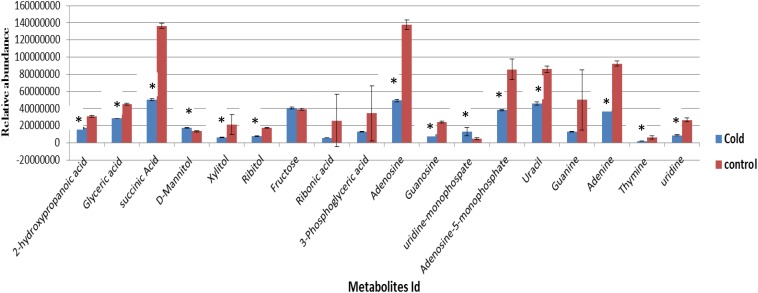
The relative abundances of cytoplasmic metabolites extracted from the cells grown to a mid-exponential phase of growth at 37°C (control, *n* = 4) compared with equivalent sets of the cells that were subsequently exposed to lower temperature of 4°C (Cold, *n* = 4) (mean ± SD, *p* < 0.05). ^∗^Significantly altered metabolites.

Eight biological replicates of *S. aureus* were grown to the mid-exponential phase of growth under ideal conditions. Four biological replicates were collected for analysis to represent the control samples to compare with the remaining four biological replicates which were incubated at 4°C before harvesting for metabolic analysis. The cell numbers for replicates harvested at 37°C were 5.7 ± 0.9 × 10^7^ and the cell numbers for cold stressed samples were 5.5 ± 0.4 × 10^7^. The results indicated that no significant reduction in cell numbers following incubation in cold temperatures (4°C). Metabolites analysis by GC-MS indicated that the cytoplasmic metabolites in cold-stressed replicates had numerous changes in the profile compositions in comparison to reference control samples (Figure 1). A significant decrease in purine and pyrimidine bases, nucleosides and organic acids abundances was observed in cells incubated at low temperature in comparison to samples grown under ideal conditions representing considerable changes in metabolic homeostasis. Among the identified carbohydrates, a significant reduction of two sugar alcohols including ribitol and xylitol was observed, but mannitol was significantly increased (Figure 1).

**FIGURE 2:**
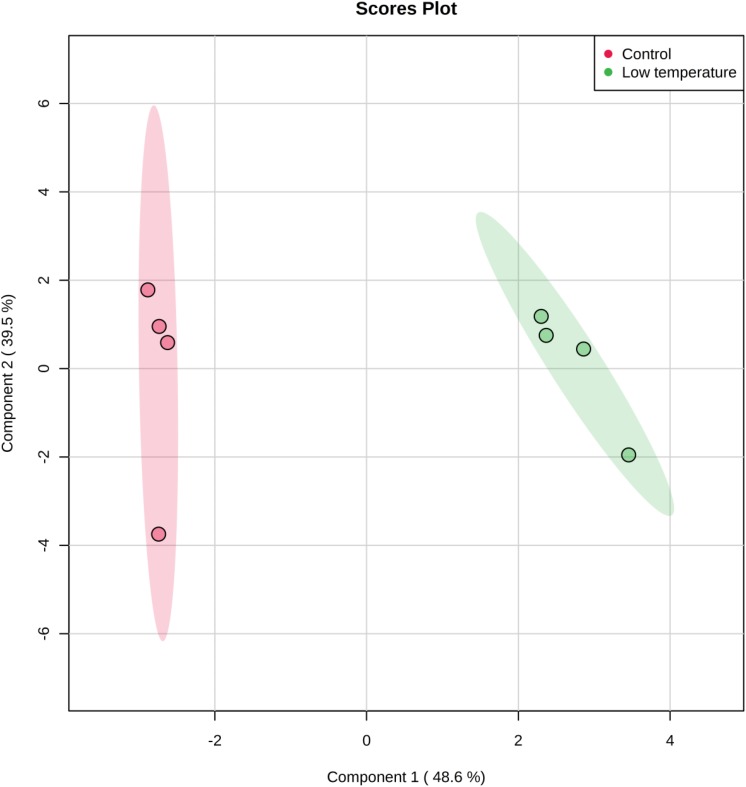
Partial Least Square-Discriminate Analysis (PLS-DA) scores (component 1 versus component 2) plotted from *S. aureus* profiles of cytoplasmic metabolites. The *S. aureus* cultures were grown under ideal conditions at 37°C (Control, *n* = 4) or exposed to low temperature at 4°C for 2 weeks (low temperature, *n* = 4) before the extraction of metabolites for analyses by GC-MS.

**FIGURE 3:**
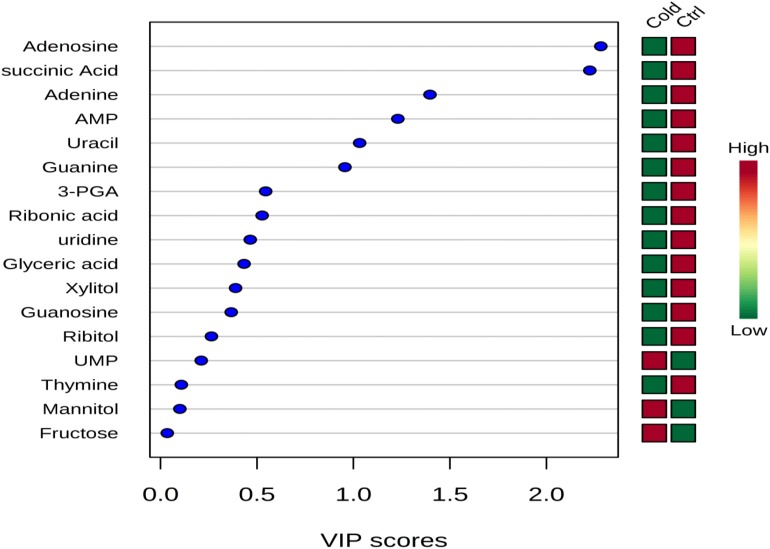
Variable importance in projection (VIP) scores indicating the top important metabolites contributing to the separation of metabolic profiles in control replicates vs. cold stressed samples. The relative abundance of metabolites is indicated by a colored scale from green to red representing the low and high, respectively.

The cytoplasmic metabolite compositions from the cells grown under optimal conditions and those exposed to lower temperature of 4°C for 2 weeks were further investigated using partial least square discriminate function (PLS-DA) to provide important insights into metabolic profiles of two groups. The PLS-DA analysis rendered a two-component model as calculated by cross-validation (CV). The PLS-DA analysis scores for control and stressed samples showed two obvious clusters separated by component 1 scores where the cytoplasmic metabolites analyzed from control samples positioned at the negative component 1 and the treatment samples positioned at the positive side of component 1 (Figure 2). Explanation of 87.4% of the analyzed data was accomplished (i.e., *R2* = 0.97 and *Q2* = 0.95) and the eigenvalue for component 1 was 14.5 as compared to 2.4 for component 2. The great variation between the eigenvalues point out that most of the explained data differences associated with clustering of the cold-stressed samples from untreated cells. In corollary, as shown in PLS-DA graph, metabolites analyzed from untreated cells were clustered separately from the cells exposed to low temperature suggesting the presence of different metabolic patterns in the two treatment regimens. The cells incubated at lower temperature of 4°C for 2 weeks were characterized via a great reduction in the abundances of measured metabolites in particular succinic acid and adenosine as shown in Figure 3.

The cytoplasmic metabolites that were contributing to the classification of control and cold-stressed samples were also identified using the variable importance in projection (VIP) scores (Figure 3). Metabolites with greater VIP scores are more important in contributing to the separation between the two groups, while those with smaller VIP scores have less influence to the model. Metabolites such as adenosine, succinic acid, adenine adenosine-mono-phosphate (AMP) had VIP score greater than 0.1 indicating that these metabolites played an essential role in the discrimination of metabolic profiles of cells grown under ideal conditions and those exposed to lower temperature of 4°C (Figure 3).

### Metabolic Enrichment and Metabolic Pathway Analyses

**FIGURE 4:**
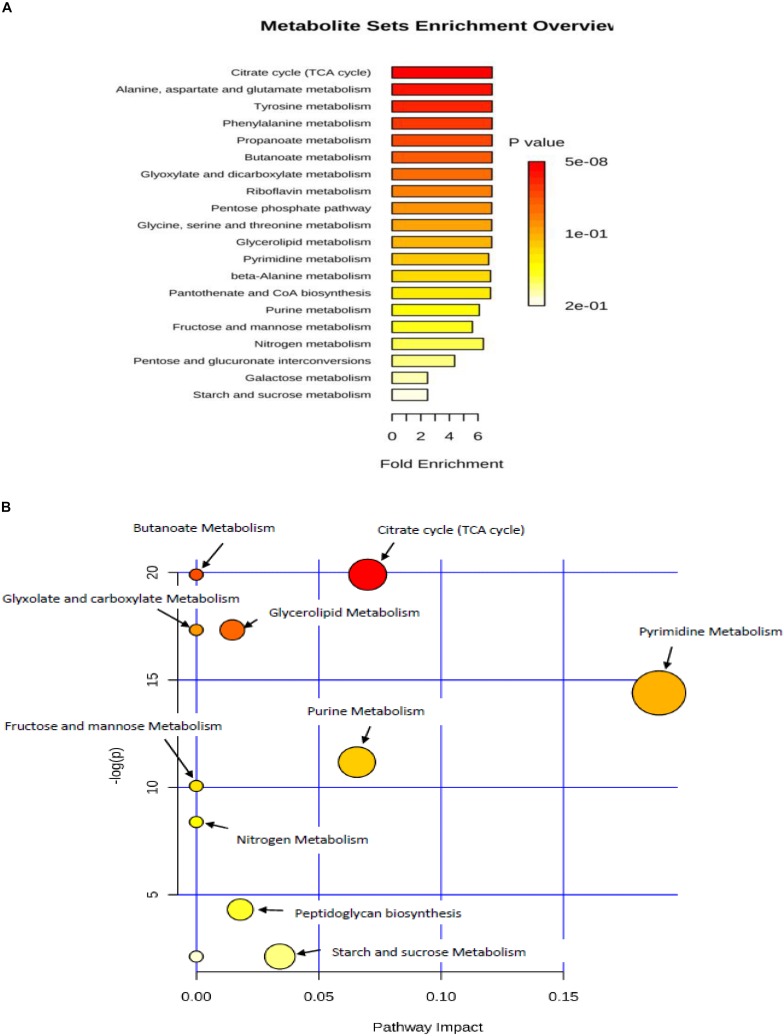
**(A)** metabolic set enrichment using metabolite concentrations showing the most enriched pathways in response to lower temperature of 4°C. **(B)** Pathway impact analysis. All the matched pathways are shown as circles. The color and size of each circle is based on *p*-value and pathway impact value, respectively. Colors of the circle based on *p*-values. The darker colors indicate more significant pathway (*p < 0.05*).

The metabolite set enrichment and metabolic pathway analyses were performed using MetaboAnalyst 4.0 to find out the most altered metabolic pathways following incubation in prolonged cold environment. The pathway impact value was calculated from topology pathway analysis, those with score higher than 0.10 was considered as a significant metabolic pathway. The analysis identified 10 matched pathways, including purine metabolism, pyrimidine metabolism, TCA cycle, butanoate metabolism, propionate metabolism, pyruvate metabolism, glycolysis and gluconeogenesis, fructose and mannose metabolism, pentose glucose interconversion, amino sugar and nucleotide sugar metabolism, sulfur metabolism, glyxolate decarboxylic metabolism, glycine serine and threonine metabolism and glycerolipid metabolism (Figure 4B). However, six metabolic pathways were significantly altered due to cold treatment, including TCA cycle, glycerolipid metabolism, pyrimidine metabolism, purine metabolism, peptidoglycan biosynthesis (Figure 4B). The quantitative enrichment analysis (MESA) was used to determine the major perturbed pathways affected in cold stressed samples (Figure 4A). Twenty biochemical pathways were identified by the analysis, seven of which were significantly affected by the cold treatment (*p* < 0.05) including citrate cycle, alanine-aspartate and glutamate metabolism, tyrosine metabolism, phenylalanine metabolism, propionate metabolism, butanoate metabolism, glyoxylate and decarboxylate metabolism (*p* < 0.05).

### Metabolites Analyses at Elevated NaCl (5%)

**FIGURE 5:**
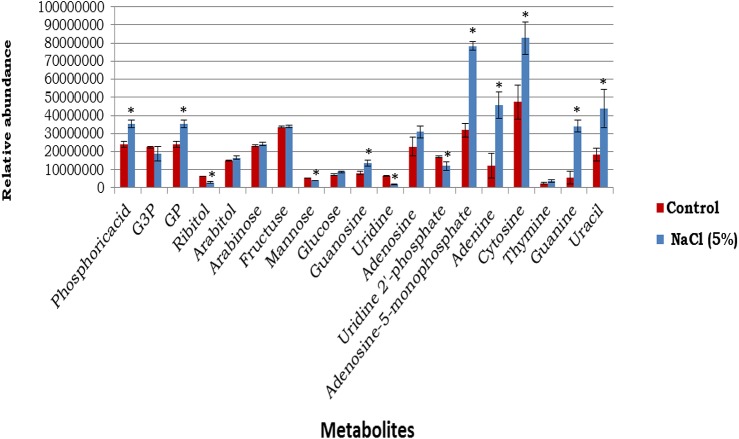
The abundances of cytoplasmic metabolites extracted from cells incubated in a defined medium (reference control, *n* = 4) compared with equivalent sets of cells that were incubated in the defined medium supplemented with 5% NaCl (*n* = 3) (mean ± SD). ^∗^*p* < 0.05. *Signicantly altered metabolites.

The number of cells was assessed by the plating techniques to determine colony forming units (CFU) at initial time of incubation for both control and NaCl stressed samples, which produced values of 5.5 ± 0.6 × 10^8^ and 5.8 ± 0.8 × 10^8^ ( ± *SD*), respectively. At the final time of incubation the cell numbers for control and NaCl stressed samples were 5.3 ± 0.5 × 10^8^ and 5.6 ± 0.9 × 10^8^ ( ± *SD*), respectively. These results suggested that no significant alterations in the cell numbers occurred over the period of incubation. The metabolic analysis of cytoplasmic extract identified 19 metabolites in both reference control and osmotic stress (5% NaCl) samples (Figure 5). Thirteen of these metabolites had undergone statistically relevant changes, whereas seven metabolites did not display any alterations in their abundance when the cells were incubated at an additional 5% NaCl, compared with reference control samples. Sugar alcohols, including ribitol, were significantly decreased in response to osmotic stress, but arabitol remained unchanged. Glucose was increased, but fructose did not show any differences among the groups. Purine and pyrimidine bases significantly increased when cells were exposed to elevated NaCl, with the exception of thymine, which remained unchanged in both groups. Guanosine levels considerably increased while uridine significantly decreased in response to osmotic stress (Figure 5).

**FIGURE 6:**
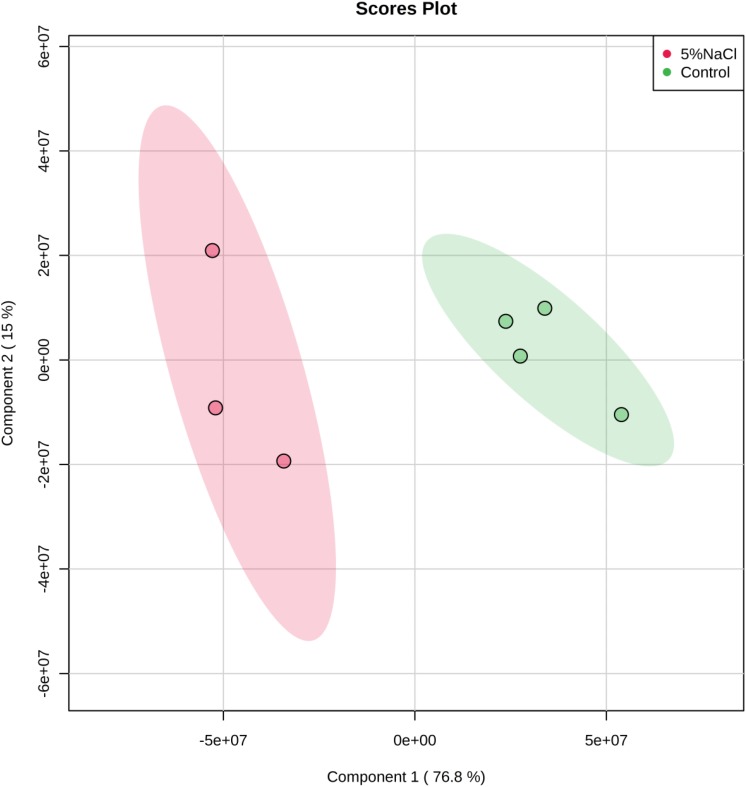
Partial least Square-discriminant analysis (PLS-DA) scores (component 1 versus component 2) plotted from *S. aureus* metabolomic data. The *S. aureus* cells were incubated in a defined minimal medium (control, *n* = 4) or with the presence of additional NaCl (5% NaCl, *n* = 3) before extraction of metabolites and analysis by GC-MS.

The cytoplasmic metabolic profiles from reference control and NaCl-treated cells were further evaluated using partial least square discriminate function (PLS-DA). The PLS-DA analysis rendered a two-component model as validated by cross-validation (CV). The PLS-DA analysis scores for cells grown under ideal conditions and those incubated with additional of 5% NaCl revealed two obvious clusters separated by component 1 scores where the cytoplasmic metabolites analyzed from control replicates placed at the positive component 1 and the NaCl-treatment samples placed at the negative side of component 1 (Figure 6). Clarification of 92.1% of the input data was achieved (i.e., *R2* = 0.93 and *Q2* = 0.85) and the eigenvalue for component 1 was 12.5 as compared to 2.8 for component 2. The great difference between the eigenvalues indicated that most of the accomplished data variations associated with clustering of the NaCl-stressed replicates from unstressed cells. In importance, as displayed in PLS-DA graph, metabolites analyzed from control cultures were gathered separately from the cells incubated with additional of 5% NaCl suggesting the presence of different metabolic profiles in the two groups. The cells treated with 5% NaCl were characterized via a general increase in the level of nitrogenous bases, while the control samples were described by an overall reduction in the metabolite levels with an exception of mannose and ribitol which were relatively increased.

**FIGURE 7:**
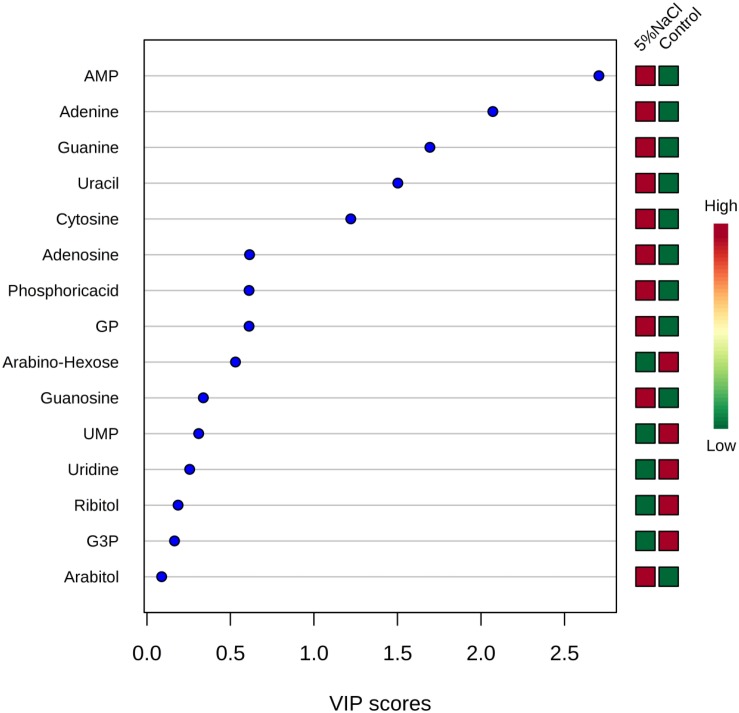
Variable importance in projection (VIP) scores for the top metabolites contributing to variation in metabolic profiles of controls vs. cold treatment samples. The relative abundance of metabolites is indicated by a colored scale from green to red representing the low and high, respectively.

Variable importance in projection analysis was conducted to determine the top metabolites contributing to the variation of metabolic profiles of control and NaCl treatment samples (Figure 7). Metabolites with a VIP score of 1.0 have been considered as significantly contributed to the differences between the two groups. As shown in Figure 7, adenosine-monophosphate (AMP), adenine, guanine, uracil and cytosine were the most metabolites providing the classification between the cells grown under ideal conditions and those incubated with additional of 5% NaCl.

### Metabolic Enrichment and Metabolic Pathway Analyses

**FIGURE 8:**
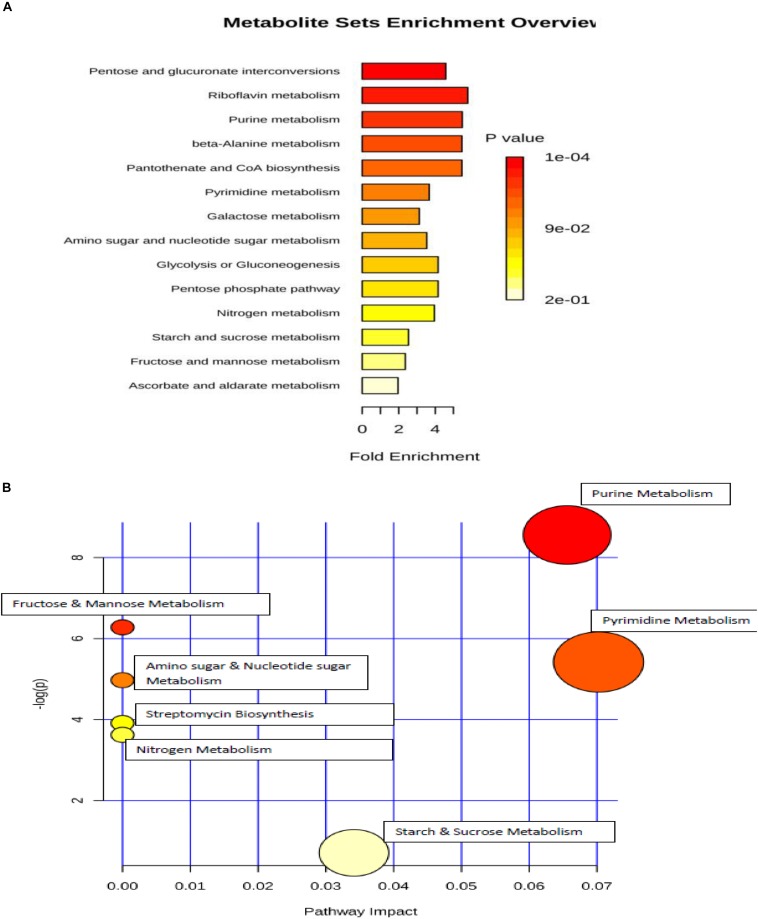
**(A)** metabolic set enrichment using metabolite concentrations showing the most altered pathways in elevated NaCl (5%). **(B)** pathway impact analysis. All the matched pathways are shown as circles. The color and size of each circle is based on *p*-value and pathway impact value, respectively. Colors of the circle based on *p*-values. The darker colors indicate more significant changes of the pathways (*p < 0.05*).

Metabolic set enrichment overview revealed that analyzed metabolites were involved in the perturbation of 14 metabolic pathways due to osmotic pressure (Figure 8A). Out of these, five pathways were found to be the most affected during the incubation in elevated NaCl (5%), including pentose and glucuronate interconversions, riboflavin metabolism, purine metabolism, beta-Alanine metabolism, pantothenate and CoA biosynthesis. Metabolic pathway analysis indicated that seven pathways were differentially regulated in response to osmotic stress (Figure 8B), two of which were significantly enriched including purine and pyrimidine metabolism.

## Discussion

The results from the present study revealed that the profiles of cytoplasmic metabolites observed in *S. aureus* grown under ideal conditions were different to those observed in cells grown under lower temperature of 4°C or elevated NaCl (5%). It was obvious that the demands for cytoplasmic metabolites altered following exposure to variations in the environmental conditions including, temperature and osmolality. Metabolites represent important precursors for the biosynthesis of nucleic acids, lipids and cell wall constituents as well as they play an important role in the metabolic homeostasis and cell integrity.

Metabolic analysis showed a general reduction in the metabolite abundances of the cells incubated at 4°C for 2 weeks compared with reference control samples grown under optimal conditions. However, the levels of these metabolites were different, which presumably reflected differential metabolic demands. Reduced metabolites in the cytoplasm of bacterial cells may represent slower metabolic rates in the cytoplasm. This would represent an essential way in adjusting the cellular homeostasis under changes in the environmental parameters. A Prior investigation revealed that *S. aureus* had reduced the levels of amino acids and glycolysis enzymes following exposure to lower temperature (Alreshidi et al., 2015). Recent study demonstrated that alterations in environmental conditions including variations in temperature and osmolality led to variations of amino acid uptake and release of *S. aureus* (Alreshidi et al., 2020).

It is not yet clear why mannitol significantly increased in cells exposed to cold stress whereas other carbohydrates either did not change or significantly reduced. Mannitol is synthesized from glucose and has been established to have an important role as an osmoprotectant in many bacteria including *S. aureus* (Edwards et al., 1981; Kets et al., 1996; Sand et al., 2013; Zahid et al., 2015). The cytoplasmic mannitol abundance was reported to play vital roles in response to high temperature and salinity, and pathogenicity in fungi (Chaturvedi et al., 1996; Calmes et al., 2013; Nguyen et al., 2019). The consumption of mannitol by *S. aureus* was shown to be essential to combat human skin antimicrobial fatty acids (Kenny et al., 2013). Also unexpected production of uridine monophosphate (UMP) was also noted in the cells stored in lower temperature for 2 weeks. This high production of UMP was possibly associated with the high requirement to synthase a thicker cell wall. A pervious study revealed that *S. aureus* had a thinker cell wall in response to prolonged cold stress compared with cells harvested at 37°C (Onyango et al., 2012, 2013).

Metabolic pathway and enrichment analyses revealed six biochemical pathways altered following exposure to lower temperature suggesting theses pathways were essential in combating the stress. Most of these metabolic pathways were involved in the energy production. The most affected metabolic pathway was citric acid cycle; this may suggest the lower energy production by cells exposed stress to minimize the energy during the stressful conditions. The substantial alteration in purine and pyrimidine pathways was represented by the significant reduction in their metabolites. The reductions in these metabolites may indicate the consumption of these metabolites to produce proteins to combat the cold stress. Our prior investigation showed that cold stress led to significant increase in nine ribosomal proteins and a substantial decrease in the majority of amino acids (Alreshidi et al., 2015). An earlier study showed that, the abundance of nucleotides was reduced ten-folds in lactic bacteria when grown in lactose medium (Kilstrup et al., 2005).

Succinic acid was one of the metabolites that greatly contributed to the classification of metabolic profiles in control and cold treatment samples as depicted by PLS-DA plot (Figure 3) which was remarkably decreased in cells exposed to cold stress. The reduction in succinic acid may characterize an effective mechanism to conserve and maintain lower metabolic levels, as it plays an important role in energy production and donation (Vandecasteele et al., 2004; Kilstrup et al., 2005). The general reduction associated with the key metabolites involving in the energy synthesis could be linked with slower metabolism which is a common mechanism used by bacteria to adapt and facilitate survival during stress (Onyango et al., 2012). It could also suggest the utilization of these metabolites in the synthesis or conversion to other compounds to develop the adaptation processes in response to environmental stresses (Dorries and Lalk, 2013).

The exposure of *S. aureus* to osmotic stress led to an increase in the majority of the identified metabolites, in particular purine and pyrimidine bases. This increase may represent a conservation and survival mechanism in response to osmotic stress, as a result of the inhibition of physiological processes, including DNA replication, that occurs due to sudden plasmolysis (Csonka, 1989). It has been suggested that purine metabolites may provide stress protection in plants (Watanabe et al., 2014). Similar results were obtained when the effects of osmotic stress was demonstrated on *Salmonella*, with significantly elevated levels of intracellular metabolites, including purines and pyrimidines (Csonka, 1989; Burgess et al., 2016). This finding is thus consistent with current findings of this study. Recently, it has been shown that considerable alterations in amino acid composition during incubation with additional 5% NaCl (Alreshidi et al., 2016, 2019), which may explain the up-regulation of nucleotide bases in this study. It has been reported that *E. coli* required producing purine and pyrimidine bases to induce infections in the mouse intestine (Vogel-Scheel et al., 2010).

The changes metabolic profiles observed in this study could indicate the existence of a phenotypic shift that can occur within a mixture of populations (Alreshidi et al., 2013). The phenotypic shifts in bacterial cells and colonies such as SCVs has been previously demonstrated following exposure to various environmental parameters including osmotic and cold stresses (Onyango et al., 2012, 2013; Crompton et al., 2014). Earlier studies have shown that SCVs have slower generation times, reduced release of virulence factors and are found to be auxotrophic for hemin and menadione compounds (Von Eiff, 2008; Proctor et al., 2014; Bui et al., 2015). Thus, it has been suggested, that bacterial cells persistently detect and respond to stress conditions by establishing the most effective and efficient phenotypes for survival. SCVs are highly associated with biofilm formation. These two lifestyles of *S. aureus* represent a very robust protective and adaptive mechanism in response to environmental threats. Metabolic studies have demonstrated a strong relationship between biofilm formation and metabolite changes. For example, it has been indicated that polysaccharide intercellular adhesin (PIA) production in biofilm is regulated by tricarboxylic acid cycle components (Sadykov et al., 2010) and the osmotic stress stimulates biofilm formation (Knobloch et al., 2001; Oniciuc et al., 2016). Several metabolites associated with purine and pyrimidine catabolism were reported to significantly contribute to distinguishing between planktonic and biofilm cells (Liebeke et al., 2011; Ammons et al., 2014). It has also been shown that the uptake of purine bases were highly necessary for biofilm formation (Zhu et al., 2007). This may explain the significant up-regulation of purine and pyrimidine nucleotides under conditions of osmotic stress in this study.

## Conclusion

The detection of cytoplasmic metabolites that differentiate between control and treatment samples may help in controlling *S. aureus* adaptation and survival. Indeed, the outcomes of this study indicated that the incubation of *S. aureus* with both cold and osmotic treatments led to significant variations in the cytoplasmic metabolite composition. Exposing the bacteria to lower temperature of 4°C resulted in a significant reduction in the majority of analyzed metabolites, whereas the cells incubated with elevated NaCl conditions had increased levels of identified metabolites. It is evident that certain strategies were developed to ensure survival during the incubation in low temperature and elevated NaCl to obtain optimal metabolism status by altering metabolite levels. It was thus concluded that these adjustments in metabolite levels may have assisted this bacterium to survive alterations in environmental conditions. More knowledge of adaptability and survival capability of *S. aureus* under various stress conditions may lead to a better understanding of sterilization methods and food storage as well as control of infection.

## Data Availability Statement

The raw data supporting the conclusions of this manuscript will be made available by the authors, without undue reservation, to any qualified researcher.

## Author Contributions

MA designed the experiments, acquired data, analyzed and interpreted data and has written the manuscript.

## Conflict of Interest

The author declares that the research was conducted in the absence of any commercial or financial relationships that could be construed as a potential conflict of interest.
